# Supplementary material for: Public Perspectives on Exposure Notification Apps: A Patient and Citizen Co-Designed Study
Source: J Pers Med. 2022 Apr 30;12(5):729. doi: 10.3390/jpm12050729 (PMC9142914; doi:10.3390/jpm12050729)
Supplement: Supplementary file 1 [file jpm-12-00729-s001.zip › jpm-1690871 - Supplementary Materials/Supplementary Document S1_Survey Questionnaire.pdf]

Questionnaire

Invitation

You are invited to participate in a research project at the Research Centre of the Centre hospitalier de l'Université de Montréal (CHUM). This project focuses on the COVID-19 exposure notification apps that can be downloaded to your smartphone. The purpose of these apps is to notify users that they may have been exposed to the virus. The questionnaire is about "COVID Alert," the app used in Quebec.

Your participation is voluntary and you can stop at any time. Your answers will be treated anonymously. The estimated response time is less than 10 minutes. Thank you for your participation!

If you have any questions or comments, please contact the researcher in charge, Dr. Marie-Pascale Pomey.

➢ marie-pascale.pomey@umontreal.ca.

Definitions

The purpose of these **smartphone apps** is to quickly notify people who have been in contact with someone who has tested positive for COVID. They are called exposure notification apps. COVID Alert is the proposed app in Quebec.

Personal data is data that identifies you, such as your name, address or other private information.

Questionnaire

Q_1 Do you have a smartphone?

1=Yes
2=No

If q#1=2->2

Q_2 If you had a smartphone, would you have downloaded the COVID Alert app?

1=Yes
2=No
9=*I don't know/I prefer not to answer

If q#1=1->3

Q_3 Did you download the COVID Alert app?

1=Yes
2=No
3=I tried to download it but it didn't work
9=*I don’t know/I prefer not to answer

If q#3=1->4

Q_4 The app is…

1=Activated
2=Not activated
3=Uninstalled
9=*I don’t know/I prefer not to answer

If q#3=1,3->5

Q_5 Who influenced you the most about downloading the app? (Check no more than two answers.)

1=Your friends or relatives
2=Your professional network
3=Health experts
4=The Quebec government
5=The Government of Canada
6=Social networks
90=Other (please specify below)
99=*I don’t know/I prefer not to answer

If q#3=1->6

Q_6 Have you ever received an alert on the COVID Alert app?

1=Yes
2=No
9=*I don’t know/I prefer not to answer

If q#3=2->7

Q_7 Why didn’t you download it?

<<______________________________>>
999=*I don’t know/I prefer not to answer

Q_8 Complete the following sentence: I would download the app... (check no more than two answers)

1=if the pandemic lasts a long time
2=if I had more contact with other people
3=if there was more evidence that it can reduce transmission of the virus
4=if there were more explanations of how it works
5=if more people were using it around me
95=*None of the above

99=*I don’t know/I prefer not to answer

Q_9 Have you tested positive for COVID-19?

1=Yes
2=No
9=*I don’t know/I prefer not to answer

If (q#9=1 et q#3=1)->10
Q_10 Did you report it in the app?

1=Yes
2=No
9=*I don’t know/I prefer not to answer

If (q#9=2,9) et (q#3=1)->11
Q_11 If you had received a positive test for COVID-19, would you report it in the app?

1=Yes
2=No
9=*I don’t know/I prefer not to answer

If (q#10=2 ou q#11=2)->12
Q_12 Why?

1=I don't know how to do it
2=I don't want to share this information
3=I didn't receive the anonymous code to do it
90=*Other (please specify below)
99=*I don’t know/I prefer not to answer

If q#3=1->13
Q_13 What do you think could be improved in the app? (Please be as specific as possible.)

<<___________________>>
999=*I don’t know/I prefer not to answer

Q_14 Complete the following sentence: The COVID-19 pandemic...

1=doesn't worry me at all
2=worries me a little bit
3=worries me moderately
4=worries me quite a bit
5=worries me a great deal
9=*I don’t know/I prefer not to answer

Q_15 Complete the following sentence: In my opinion, my risk of exposure to COVID-19 is...

1=very low
2=low
3=moderate
4=high
5=very high
9=*I don’t know/I prefer not to answer

Q_16 Complete the following sentence: My knowledge of the COVID Alert app is...

1=very low
2=low
3=average
4=high
5=very high
9=*I don’t know/I prefer not to answer

Q_17 Do you think the application can identify users?

1=Yes
2=No
9=*I don’t know/I prefer not to answer

Q_18 Do you think the application locates users by GPS?

1=Yes
2=No
9=*I don’t know/I prefer not to answer

Q_19 In your opinion, does this app help fight the COVID-19 pandemic?

1=Yes
2=No
9=*I don’t know/I prefer not to answer

Q_20 Do you think an app like COVID Alert should be used for other health emergencies?

1=Yes
2=No
9=*I don’t know/I prefer not to answer

Q_21 In your opinion, does the app protect the people most vulnerable to COVID-19 (e.g., the elderly, immunocompromised people)?

1=Yes
2=No
9=*I don’t know/I prefer not to answer

Q_22 In your opinion, does the app protect your personal data adequately?

1=Yes
2=No
9=*I don't know/I prefer not to answer

Q_23 Do you have any concerns about this type of app?

1=Yes
2=No
9=*I don't know/I prefer not to answer

If q#23=1->24
Q_24 What is your main concern? (Please be as specific as possible.)

<<________________>>
999=*I don't know/I prefer not to answer

Q_25 To what extent do you agree with the following statements about the COVID Alert app?

The information collected by the app could be used to monitor the population.

1=Completely disagree
2=Disagree
3=Agree
4=Completely agree
9=*I don't know/I prefer not to answer

Q_26 The app only collects the information that I have consented to provide.

1=Completely disagree
2=Disagree
3=Agree
4=Completely agree
9=*I don't know/I prefer not to answer

Q_27 The app can cause unnecessary stress.

1=Completely disagree
2=Disagree
3=Agree
4=Completely agree
9=*I don't know/I prefer not to answer

Q_28 The app may unnecessarily clog up the health care system (e.g., by pushing non-infected people to get tested).

1=Completely disagree
2=Disagree

3=Agree
4=Completely agree
9=*I don’t know/I prefer not to answer

Q_29 Do you agree with the following statement?

If I receive an exposure alert, the COVID Alert app should help me schedule a COVID-19 screening test.

1=Completely disagree
2=Disagree
3=Agree
4=Completely agree
9=*I don’t know/I prefer not to answer

Q_30 Do you agree with the following statement?

With my permission, the COVID Alert application should share some anonymous data with the anonymous data with the Quebec health network (e.g. if I receive a positive diagnosis or an exposure alert).

1=Completely disagree
2=Disagree
3=Agree
4=Completely agree
9=*I don’t know/I prefer not to answer

Q_31 To what extent do you agree with the following statement about the COVID Alert app?

In a health emergency like the COVID-19 pandemic, this type of app should be mandatory for the population.

1=Completely disagree
2=Disagree
3=Agree
4=Completely agree
9=*I don’t know/I prefer not to answer

If q#31=1,2,9->32

Q_32 To what extent do you agree with the following statement about the COVID Alert app?

This type of application should be mandatory for the population if there was a health emergency more dangerous and deadly than the COVID-19 pandemic.

1=Completely disagree
2=Disagree
3=Agree

4=Completely agree
9=*I don’t know/I prefer not to answer

Q_33 To what extent do you agree with the following statements about the COVID Alert app?

This type of app should be mandatory for the population to have access to certain places or services (e.g. CHSLD, hospitals, schools).

1=Completely disagree
2=Disagree
3=Agree
4=Completely agree
9=*I don’t know/I prefer not to answer

Q_34 This type of app should be mandatory for some group of people (e.g. health professionals, teachers).

Q_35 Citizens should be involved in the development of this type of app.

Q_36 Would you be interested in participating in the development of this type of app?

1=Yes
2=No
9=*I don’t know/I prefer not to answer
