# Supplementary material for: Public Perspectives on Exposure Notification Apps: A Patient and Citizen Co-Designed Study
Source: J Pers Med. 2022 Apr 30;12(5):729. doi: 10.3390/jpm12050729 (PMC9142914; doi:10.3390/jpm12050729)
Supplement: Supplementary file 1 [file jpm-12-00729-s001.zip › jpm-1690871 - Supplementary Materials/Supplementary Document S2_R citations.pdf]

Citation

1. R Core Team (2022). R: A language and environment for statistical computing. R Foundation for Statistical Computing, Vienna, Austria. URL https://www.R-project.org/.

Version

2. "R version 4.1.3 (2022-03-10)"
   Packages
3. Hadley Wickham and Jennifer Bryan (2022). readxl: Read Excel Files. R package version 1.4.0. https://CRAN.R-project.org/package=readxl
4. T. Lumley (2010) Complex Surveys: A Guide to Analysis Using R. John Wiley and Sons.
5. Hadley Wickham, Romain François, Lionel Henry and Kirill Müller (2022). dplyr: A Grammar of Data Manipulation. R package version 1.0.8. https://CRAN.R-project.org/package=dplyr
6. Max Gordon and Thomas Lumley (2021). forestplot: Advanced Forest Plot Using 'grid' Graphics. Rpackage version 2.0.1. https://CRAN.R-project.org/package=forestplot
7. Thomas Lumley (2021). svyVGAM: Design-Based Inference in Vector Generalised Linear Models. Rpackage version 1.0. https://CRAN.R-project.org/package=svyVGAM
8. Ben-Shachar M, Lüdecke D, Makowski D (2020). effectsize: Estimation of Effect Size Indices and Standardized Parameters. Journal of Open Source Software, 5(56), 2815. doi:10.21105/joss.02815
9. Julien Barnier, François Briatte and Joseph Larmarange (2022). questionr: Functions to Make Surveys Processing Easier. R package version 0.7.7. https://CRAN.R-project.org/package=questionr
10. Dane R. Van Domelen (2021). tab: Create Summary Tables for Statistical Reports. R package version 5.1.1. https://CRAN.R-project.org/package=tab

Link of the code in OSF.

The folder is public. https://osf.io/dwfhy/
