# Supplementary material for: Public Perspectives on Exposure Notification Apps: A Patient and Citizen Co-Designed Study
Source: J Pers Med. 2022 Apr 30;12(5):729. doi: 10.3390/jpm12050729 (PMC9142914; doi:10.3390/jpm12050729)
Supplement: Supplementary file 1 [file jpm-12-00729-s001.zip › jpm-1690871 - Supplementary Materials/Supplementary Document S3_Model specification.pdf]

Supplementary file S3

Methodology

To assess the determinants of the download of the COVID Alert app, a multivariate logistic regression model was estimated. The logistic model captures the probability of the download of the COVID alert app and the individual effect of socioeconomical and opinion and perception variables. For the analysis we included only adults who own a smartphone and the observations that indicated “I don’t know/I prefer not to answer” were excluded. In addition, individuals for whom we could not identify the region of residency were excluded.

In our model we view the download as dependent variable and socioeconomical characteristics, perceptions of the pandemic, app literacy, and opinions and perceptions of the COVID Alert app as predictors. The variables included in the model responded to what was found in the literature.

The model takes the general form:

$$logit\ [\pi(X)] = \beta_0 + \beta_1 X_1 + \beta_2 X_2 + \cdots \beta_j X_j$$

where $\pi(X)$ is a binary variable that takes the values one or zero if the individual downloaded the app or not, respectively. $X_1 \cdots X_j$ are the predictor variables and $\beta_1 \cdots \beta_j$ are the parameters of interest.

The model yields the results in the form of log-odds but for interpretability we calculated the inverse of these to obtain the odds-ratio. Table 1 Results of regression analyses on the download of the COVID Alert app. Table 1 presents the results of the regression analyses for the download of the COVID Alert app.

Table S1 Results of regression analysis on the download of the COVID Alert app

**Download Covid Alert App. Reference: did not**	**Odd ratio**	**Std. Error**	**95% Confidence interval**	
(Intercept)	2.06	1.52	0.10	40.83
**Gender (Ref: Male)**				
Female	2.26**	0.26	1.36	3.76
Gender diverse	8408714***	1.318336	631984	1.12E+08
**Age (Ref: 18-24 years)**				
25-34	2.18	0.88	0.39	12.16
35-44	1.06	0.85	0.20	5.69
45-54	1.01	0.87	0.18	5.54
55-64	2.50	0.89	0.44	14.35
65 over	4.09	0.97	0.61	27.43
**Region (Ref: Greater Quebec City region)**				
Greater Montreal area	0.54	0.49	0.21	1.40
Elsewhere in Quebec	0.63	0.50	0.24	1.69
**Education level (Ref: none)**				
< two year university diploma	0.99	0.88	0.18	5.57
Two year university diploma	1.39	0.89	0.24	7.96

> Two year university diploma	1.40	0.89	0.25	8.01
Other	0.00***	1.84	0.00	0.00
**Occupation (Ref: Working full time)**				
Retired	0.56	0.46	0.23	1.36
Student	0.71	0.90	0.12	4.14
Not employed	0.11**	0.68	0.03	0.43
Other	0.20*	0.78	0.04	0.92
**Primary language (Ref: English)**				
French	0.41*	0.38	0.20	0.87
Other	0.27	0.71	0.07	1.12
**Concern about the pandemic (ref: does not worry)**				
Moderate	0.73	0.53	0.26	2.07
High	0.84	0.56	0.28	2.51
Do not know/prefer not to answer	0.00***	2.26	0.00	0.00
**Individual Risk of exposure to COVID-19 (Ref: Low)**				
Moderate	1.50	0.26	0.89	2.52
High	1.61	0.56	0.54	4.85
Do not know/prefer not to answer	0.13*	0.93	0.02	0.81
**Individual knowledge of the COVID alert app (Ref: low)**				
Average	3.49***	0.29	1.98	6.14
High	5.27***	0.41	2.35	11.82
Do not know/prefer not to answer	0.00***	0.73	0.00	0.00
**App can identify users: (Ref: Agree)**				
Disagree	1.29	0.33	0.68	2.46
Do not know/prefer not to answer	0.80	0.38	0.38	1.71
**App locates users by GPS (Agree)**				
Disagree	1.03	0.33	0.54	1.95
Do not know/prefer not to answer	1.14	0.37	0.55	2.36
**App help fight the COVID-19 pandemic (Ref: Agree)**				
Disagree	0.76	0.38	0.36	1.59
Do not know/prefer not to answer	0.49	0.42	0.21	1.13
**Do you think an app like COVID Alert should be used for other health emergencies? (Ref: Agree)**				
Disagree	0.24***	0.38	0.11	0.50
Do not know/prefer not to answer	0.37**	0.36	0.18	0.76
**The app protects the people most vulnerable to COVID-19 (Ref: Agree)**				
Disagree	1.65	0.32	0.88	3.08

Do not know/prefer not to answer	1.55	0.53	0.55	4.37
**The app protects your personal data (Ref: Agree)**				
Disagree	0.54	0.41	0.24	1.22
Do not know/prefer not to answer	0.86	0.34	0.44	1.69
**Have concerns about this type of app (Ref: Agree)**				
Disagree	1.25	0.34	0.65	2.42
Do not know/prefer not to answer	0.48	0.57	0.16	1.49
**The information collected by the app could be used to monitor the population (Ref: Disagree)**				
Agree	0.92	0.32	0.49	1.71
Do not know/prefer not to answer	0.47	0.54	0.16	1.36
**The app only collects the information that I have consented to provide (Ref: Disagree)**				
Agree	2.28*	0.39	1.06	4.89
Do not know/prefer not to answer	1.14	0.48	0.44	2.94
**The app can cause unnecessary stress (Ref: Disagree)**				
Agree	0.25***	0.29	0.14	0.45
Do not know/prefer not to answer	0.44	0.66	0.12	1.62
**The app may unnecessarily clog up the health care system (Ref: Disagree)**				
Agree	0.29***	0.35	0.15	0.57
Do not know/prefer not to answer	1.01	0.53	0.35	2.88
**Usage frequency of common mobile apps (Ref: Often)**				
Sometimes	0.32**	0.43	0.14	0.76
Never	0.00***	0.71	0.00	0.00
Do not know/prefer not to answer	0.32	0.99	0.05	2.21

*** P<0.001, **P<0.01, *P<0.05

As an exploratory analysis of the relation of the citizens' perceptions on the app technical characteristics and preoccupations variables we estimated 2 additional logistic models. Table 2 and 3 present the results of the regression analyses of these models.

Table S2 Results of regression analysis on the perception that the app can identify users

**App can identify users. Reference (Ref): No**	**Odd ratio**	**Std. Error**	**95% Confidence interval**	
(Intercept)	1.06	1.57	0.05	23.03
**Gender (Ref: Male)**				
Female	1.21	0.26	0.73	2.02
Gender diverse	0.00***	1.40	0.00	0.00
**Age (Ref: 18-24 years)**				
25-34	4.55*	0.76	1.02	20.28
35-44	1.42	0.80	0.30	6.83
45-54	4.23	0.77	0.94	19.10
55-64	2.71	0.79	0.57	12.83
65 over	3.30	0.83	0.65	16.81
**Region (Ref: Greater Quebec City region)**				
Greater Montreal area	0.87	0.41	0.39	1.93
Elsewhere in Quebec	1.28	0.39	0.59	2.77
**Occupation (Ref: Working full time)**				
Retired	1.57	0.40	0.72	3.43
Student	2.78	0.71	0.69	11.22
Not employed	0.31	0.84	0.06	1.61
Other	0.66	0.70	0.16	2.60
**Education level (Ref: none)**				
< two year university diploma	0.18	1.03	0.02	1.34
Two year university diploma	0.22	1.05	0.03	1.70
> Two year university diploma	0.16	1.04	0.02	1.24
Other	1003328***	1.56	46462.52	21666240
**Primary language (Ref: English)**				
French	0.79	0.37	0.38	1.65
Other	3.08	0.80	0.63	14.94
**Individual knowledge of the COVID alert app (Ref: low)**				
Average	1.18	0.28	0.68	2.05
High	0.76	0.37	0.37	1.56
Do not know/prefer not to answer	4125705***	1	1174034	14498250
**App locates users by GPS (Agree)**				

Disagree	0.26***	0.31	0.14	0.48
Do not know/prefer not to answer	1.12	0.37	0.55	2.30
**The app protects your personal data (Ref: Agree)**				
Disagree	1.48	0.41	0.66	3.29
Do not know/prefer not to answer	0.89	0.37	0.43	1.82
**Have concerns about this type of app (Ref: Agree)**				
Disagree	0.89	0.32	0.47	1.66
Do not know/prefer not to answer	0.85	0.85	0.16	4.51
**The information collected by the app could be used to monitor the population (Ref: Disagree)**				
Agree	3.27***	0.29	1.84	5.82
Do not know/prefer not to answer	2.32	0.58	0.74	7.29
**The app only collects the information that I have consented to provide (Ref: Disagree)**				
Agree	0.49	0.40	0.22	1.08
Do not know/prefer not to answer	0.90	0.46	0.36	2.20
**Usage frequency of common mobile apps (Ref: Often)**				
Sometimes	1.19	0.33	0.62	2.27
Never	0.79	0.82	0.16	3.93
Do not know/prefer not to answer	0.47	1.31	0.04	6.09

*** P<0.001, **P<0.01, *P<0.05

Table S3 Results of the regression analysis on the perception that the app protects personal data

**The app protects your personal data. Reference (Ref): No**	**Odd ratio**	**Std. Error**	**95% Confidence interval**	
(Intercept)	0.48	2.00	0.01	24.46
**Gender (Ref: Male)**				
Female	0.52	0.38	0.25	1.09

Gender diverse	6332675***	1.52	321339	124798900
**Age (Ref: 18-24 years)**				
25-34	0.15*	0.85	0.03	0.80
35-44	0.11**	0.80	0.02	0.53
45-54	0.34	0.87	0.06	1.88
55-64	0.31	0.87	0.06	1.70
65 over	0.21	0.97	0.03	1.40
**Region (Ref: Greater Quebec City region)**				
Greater Montreal area	0.46	0.55	0.16	1.38
Elsewhere in Quebec	0.54	0.56	0.18	1.60
**Education level (Ref: none)**				
< two year university diploma	1.01	0.66	0.28	3.65
Two year university diploma	0.73	0.72	0.18	3.00
> Two year university diploma	1.02	0.81	0.21	4.99
Other	1.61	1.02	0.22	11.87
**Occupation (Ref: Working full time)**				
Retired	0.66	1.78	0.02	21.77
Student	0.53	1.82	0.01	18.99
Not employed	1.69	1.85	0.04	63.76
Other	61583610***	2.376	578169	6559577000
**Primary language (Ref: English)**				
French	0.68	0.47	0.27	1.73
Other	0.68	1.15	0.07	6.52
**Individual knowledge of the COVID alert app (Ref: low)**				
Average	3.07**	0.41	1.37	6.90
High	1.96	0.49	0.75	5.11
Do not know/prefer not to answer	0.00***	1.16	0.00	0.00
**App can identify users: (Ref: Agree)**				
Disagree	1.51	0.51	0.55	4.14
Do not know/prefer not to answer	0.42	0.86	0.08	2.30
**App locates users by GPS (Agree)**				
Disagree	1.48	0.50	0.56	3.94
Do not know/prefer not to answer	3.86	0.75	0.88	16.99
**Have concerns about this type of app (Ref: Agree)**				
Disagree	14.05***	0.41	6.23	31.70
Do not know/prefer not to answer	1.61	0.86	0.29	8.79

**The information collected by the app could be used to monitor the population (Ref: Disagree)**				
Agree	0.25***	0.39	0.12	0.54
Do not know/prefer not to answer	0.11	1.14	0.01	1.03
**The app only collects the information that I have consented to provide (Ref: Disagree)**				
Agree	24.91***	0.52	8.91	69.62
Do not know/prefer not to answer	9.56**	0.77	2.09	43.68
**Usage frequency of common mobile apps (Ref: Often)**				
Sometimes	0.56	0.58	0.18	1.74
Never	2.78	0.77	0.62	12.53
Do not know/prefer not to answer	0.00	1.36	0.00	0.00

*** P<0.001, **P<0.01, *P<0.05
