# Supplementary material for: Public Perspectives on Exposure Notification Apps: A Patient and Citizen Co-Designed Study
Source: J Pers Med. 2022 Apr 30;12(5):729. doi: 10.3390/jpm12050729 (PMC9142914; doi:10.3390/jpm12050729)
Supplement: Supplementary file 1 [file jpm-12-00729-s001.zip › jpm-1690871 - Supplementary Materials/Supplementary Document S4_Detailed methodology.pdf]

Annexe 1

MÉTHODOLOGIE DÉTAILLÉE

Cette annexe présente tous les renseignements pertinents concernant le déroulement de l'étude. Elle contient la méthodologie détaillée et les résultats administratifs du sondage, de façon à bien préciser les limites de l'étude et la portée des résultats et, le cas échéant, à reproduire l'étude selon le même protocole de recherche.

PLAN DE SONDAGE

Population cible

Collecte web

Québécois âgés de 15 ans ou plus.

Collecte téléphonique

Adultes (18 ans ou plus) québécois peu ou pas familiers avec internet.

Base de sondage

Collecte web

- Le panel Or web de SOM. Il s'agit d'un panel probabiliste constitué d'internautes recrutés aléatoirement dans le cadre de nos sondages téléphoniques auprès de la population adulte en général. Afin d'obtenir des répondants âgés de 15 à 17 ans, à la fin du questionnaire il était demandé aux parents d'enfants de cette tranche d'âge s'ils acceptaient qu'ils répondent au sondage. Le cas échéant, une invitation par courriel leur était envoyée.
- Pour obtenir davantage de répondants âgés de 15 à 17 ans, nous avons eu recours a un panel externe non probabiliste.

Collecte téléphonique

- Le panel Or téléphonique de SOM. Il s'agit d'un panel probabiliste constitué de personnes recrutées aléatoirement dans le cadre de nos sondages téléphoniques auprès de la population adulte en général.
- L'échantillon initial a été généré en ciblant les personnes qui n'utilisent pas internet.

Plan d'échantillonnage

Collecte web

- Le plan d'échantillonnage visait à obtenir 850 adultes âgés de 18 ans ou plus. Un échantillon de membres du panel Or a été tiré à l'aide d'un algorithme visant la meilleure représentativité possible en fonction de la région, de l'âge, du sexe, de la langue maternelle, de la scolarité et de la taille du ménage.
- Le plan d'échantillonnage visait aussi à obtenir 250 répondants âgés de 15 à 17 ans.

Collecte téléphonique

- Un échantillon de membres du panel Or a été tiré afin d'obtenir des adultes moins familiers avec internet.

Au total, le sondage compte **1 196 répondants**, répartis de la façon suivante :

- 15 à 17 ans : 237 répondants (résultats administratifs détaillés non présentés car presque tous les répondants proviennent d'un panel externe utilisant un recrutement non probabiliste)
- 18 ans ou plus : 959 répondants

MÉTHODOLOGIE DÉTAILLÉE (SUITE)

QUESTIONNAIRE

Le questionnaire a été élaboré par SOM, en collaboration avec le client, puis révisé, traduit en anglais et programmé par SOM.

La version finale du questionnaire est présentée en annexe.

Collecte web
- Le questionnaire était disponible en français et en anglais.
- La durée moyenne pour remplir le questionnaire se chiffre à 6,4 minutes et la médiane, à 5,7 minutes.

Collecte téléphonique
- Les entrevues ont été réalisées en français ou en anglais.
- La durée moyenne des entrevues s'est établie à 9,9 minutes.

COLLECTE

Période de collecte

Collecte web

Du 27 mai au 15 juin 2021.

Collecte téléphonique

Du 8 au 28 juin 2021.

Mode de collecte

Collecte web
- Questionnaire autoadministré par internet.
- Invitations par courriel gérées par SOM.
- Collecte web sur les serveurs de SOM.

Collecte téléphonique
- Téléphonique assistée par ordinateur.
- Gestion informatisée des numéros de téléphone.
- Gestion de l'échantillon visant des objectifs de taux de réponse supérieurs.
- Maximum de 7 appels.

Résultats administratifs de la collecte

Collecte web

Les résultats détaillés sont présentés à la page suivante. Le taux de réponse s'élève à **38 %**.

Collecte téléphonique (les résultats détaillés sont présentés à la page 5)

Taux de non-réponse	30,8 %
Taux de refus	40,7 %
**Taux de réponse**	**28,5 %**

Collecte web
RÉSULTATS ADMINISTRATIFS DÉTAILLÉS
(Adultes 18 ans ou plus)

CALCUL DU TAUX DE RÉPONSE			
**RÉSULTATS DE L'ENQUÊTE WEB**		Courriel indiquant refus de répondre	0
Taille de l'échantillon	**2 428**	Désabonnement	1
Nombre d'entrevues visées	850	Entrevue rejetée à la suite du contrôle qualité	0
**INVITATIONS ENVOYÉES**		**UNITÉ JOINTE TOTALE (C)**	931
Invitations envoyées **(A)**	2 249	**UNITÉ NON JOINTE**	
Adresse de courriel sur la liste noire	26	Serveur de courriel distant ne répond pas ou en problème	0
Échec lors de l'envoi du courriel	0	Quota de l'usager dépassé	0
**UNITÉ JOINTE RÉPONDANTE**		Détection par antipourriels	0
Questionnaires complétés	859	Autres messages de retour non reconnus	1
Hors de la population visée	0	Unité non jointe totale **(D)**	1
Accès lorsqu'un quota est atteint	0	**UNITÉ INEXISTANTE**	
Unité jointe répondante totale **(B)**	859	Courriel invalide (usager@)	1
**UNITÉ JOINTE TARDIVE**		Courriel invalide (@domaine)	0
Accès lorsque collecte de la strate terminée	21	Duplicata	0
Accès lorsque collecte terminée	0	Unité inexistante totale **(E)**	1
**UNITÉ JOINTE NON RÉPONDANTE**		**TAUX D'ACCÈS (C/(A-E))**	**41,4 %**
Abandon durant le questionnaire	50	**TAUX DE RÉPONSE PARMI UNITÉ JOINTE (B/C)**	**92,3 %**
Courriel automatique (absence du répondant)	0	**TAUX DE RÉPONSE (B/(A-E))**	**38,2 %**

MÉTHODOLOGIE DÉTAILLÉE (SUITE)

Collecte téléphonique

RÉSULTATS ADMINISTRATIFS DÉTAILLÉS
(Adultes 18 ans ou plus)

CALCUL DU TAUX DE RÉPONSE

**A)**	**ÉCHANTILLON DE DÉPART**	844
**B)**	**NUMÉROS NON VALIDES**	**91**
B1)	Hors service	86
B2)	Non résidentiel	1
B3)	Hors strate	2
B4)	Télécopieur	0
B5)	Duplicata	2
**C)**	**UNITÉS NON JOINTES (NON RÉSOLUES)**	**196**
C1)	Pas de réponse	68
C2)	Ligne occupée	6
C3)	Répondeur	91
C4)	Ligne en dérangement	31
**D)**	**UNITÉS JOINTES NON RÉPONDANTES**	**346**
D1)	Incapacité, maladie, problème de langue	22
D2)	Répondant sélectionné pas disponible	23
D3)	Refus du ménage	25
D4)	Refus de la personne	204
D5)	Refus sur cellulaire	59
D6)	Incomplet	13
**E)**	**UNITÉS JOINTES RÉPONDANTES**	**211**
E1)	Autres langues	1
E2)	Non-admissibles	110
E3)	Entrevues complétées	100

F)	Numéros non joints (C1 + C2 + C4)	105
G)	Numéros joints (A - F)	739
H)	Numéros joints non valides (B)	91
I)	Numéros joints valides (G - H)	648
J)	Estimation du nombre de numéros non joints valides (F X I ÷ G)	92
K)	Estimation du nombre total de numéros valides (I + J)	740

TAUX DE RÉPONSE ESTIMÉ DE SOM (TRE)

Non-réponse estimée (C3 + D2 + J + D1) ÷ K	30,8 %
Refus ((D3 + D4 + D5 + D6) ÷ K)	40,7 %
**TAUX DE RÉPONSE ESTIMÉ** (E1 + E2 + E3) ÷ K	28,5 %

TAUX DE RÉPONSE DE L'ARIM*

Taux de réponse (E ÷ (C + D + E))	28,0 %

*La différence entre les deux taux de réponse tient au fait que le taux de l'Association de la recherche et de l'intelligence marketing (ARIM) suppose que tous les numéros non joints sont valides alors que le taux SOM suppose plutôt qu'il y a la même proportion de numéros valides parmi les non joints que parmi ceux qui ont été joints.

PONDÉRATION ET TRAITEMENT

Pondération adultes
La pondération a été effectuée sur la base des adultes, en tenant compte des variables énumérées ci-dessous :

- La distribution conjointe d'âge (18-34 ans, 35-44 ans, 45-54 ans, 55-64 ans, 65 ans ou plus), de la région (RMR de Montréal, RMR de Québec, ailleurs au Québec) et de sexe;
- La langue maternelle (français seulement, anglais/autre);
- Le plus haut diplôme ou certificat détenu (aucun/secondaire/DEP, collégial, universitaire);
- La proportion d'internautes par région.

Pondération jeunes âgés de 15 à 17 ans
La pondération a été effectuée sur la base des jeunes âgés de 15 à 17 ans, en tenant compte de la distribution conjointe du sexe et de la région (RMR de Montréal, RMR de Québec, ailleurs au Québec)

Données de population utilisées pour l'étude :

- Institut de la Statistique du Québec 2020 (pour les distributions d'âge et de sexe)
- Recensement de 2016 (pour toutes les autres distributions)

Méthode : une pondération multivariée à 10 itérations par la méthode itérative du quotient est réalisée pour assurer une représentation fidèle à toutes ces distributions.

Les données ont été traitées à l'aide du progiciel spécialisé MACTAB. Les résultats pour chacune des questions sont présentés en fonction d'une bannière qui inclut les variables pertinentes à l'analyse des résultats.

MARGES D'ERREUR

Comme une certaine proportion de l'échantillon web a été générée de manière non probabiliste (particulièrement pour les 15-17 ans), les marges d'erreur présentées aux pages suivantes sont présentées à titre indicatif seulement. Elles sont calculées au niveau de confiance de 95 % et tiennent compte de l'effet de plan.

L'effet de plan apparaît lorsque les entrevues complétées ne sont pas réparties proportionnellement à la population d'origine selon les variables de segmentation ou de pondération. L'effet de plan est le ratio entre la taille de l'échantillon et la taille d'un échantillon aléatoire simple de même marge d'erreur. C'est une statistique utile à l'estimation des marges d'erreur pour des sous-groupes de répondants. Par exemple, au tableau de la page suivante, la marge d'erreur est la même que pour un échantillon aléatoire simple de taille 656 (959 ÷ 1,461).

Le tableau de la page suivante affiche les marges d'erreur de l'étude (en tenant compte de l'effet de plan) selon la valeur de la proportion estimée.

MÉTHODOLOGIE DÉTAILLÉE (SUITE)

ADULTES DE 18 ANS OU PLUS
MARGE D'ERREUR SELON LA PROPORTION ESTIMÉE

		RMR		
	**Ensemble**	**Grande région de Québec**	**Grande région de Montréal**	**Ailleurs au Québec**
NOMBRE D'ENTREVUES	959	122	484	353
EFFET DE PLAN	1,461	1,363	1,502	1,401
PROPORTION :				
99 % ou 1 %	0,8 %	2,1 %	1,1 %	1,2 %
95 % ou 5 %	1,7 %	4,5 %	2,4 %	2,7 %
90 % ou 10 %	2,3 %	6,2 %	3,3 %	3,7 %
80 % ou 20 %	3,1 %	8,3 %	4,4 %	4,9 %
70 % ou 30 %	3,5 %	9,5 %	5,0 %	5,7 %
60 % ou 40 %	3,7 %	10,2 %	5,3 %	6,0 %
**50 % (MARGE MAXIMALE)**	3,8 %	10,4 %	5,5 %	6,2 %

* La marge d'erreur varie selon la valeur de la proportion estimée : elle est plus grande lorsque la proportion est voisine de 50 % et plus petite à mesure que la proportion s'éloigne de 50 %.

JEUNES DE 15 À 17 ANS
MARGE D'ERREUR SELON LA PROPORTION ESTIMÉE

		RMR		
	Ensemble	Grande région de Québec	Grande région de Montréal	Ailleurs au Québec
NOMBRE D'ENTREVUES	237	41	115	81
EFFET DE PLAN	1,414	1,468	1,078	1,719
PROPORTION :				
99 % ou 1 %	1,5 %	3,7 %	1,9 %	2,8 %
95 % ou 5 %	3,3 %	8,1 %	4,1 %	6,2 %
90 % ou 10 %	4,5 %	11,1 %	5,7 %	8,6 %
80 % ou 20 %	6,1 %	14,8 %	7,6 %	11,4 %
70 % ou 30 %	6,9 %	17,0 %	8,7 %	13,1 %
60 % ou 40 %	7,4 %	18,2 %	9,3 %	14,0 %
**50 % (MARGE MAXIMALE)**	7,6 %	18,5 %	9,5 %	14,3 %

* La marge d'erreur varie selon la valeur de la proportion estimée : elle est plus grande lorsque la proportion est voisine de 50 % et plus petite à mesure que la proportion s'éloigne de 50 %.
